# Feasibility of a Mobile Phone App to Promote Adherence to a Heart-Healthy Lifestyle: Single-Arm Study

**DOI:** 10.2196/12679

**Published:** 2019-04-19

**Authors:** Pernille Lunde, Birgitta Blakstad Nilsson, Astrid Bergland, Asta Bye

**Affiliations:** 1 Department of Physiotherapy Faculty of Health Sciences Oslo Metropolitan University Oslo Norway; 2 Section for Physiotherapy Division of Medicine Oslo University Hospital Oslo Norway; 3 Department of Nursing and Health Promotion Faculty of Health Sciences Oslo Metropolitan University Oslo Norway; 4 European Palliative Care Research Centre, Department of Oncology Oslo University Hospital and Institute of Clinical Medicine University of Oslo Oslo Norway

**Keywords:** mHealth, eHealth, mobile phone app, cardiac rehabilitation

## Abstract

**Background:**

Long-term maintenance of preventive activities is fundamental for achieving improved outcomes in cardiac rehabilitation (CR). Despite this, it has been shown to be a major challenge for many patients to follow recommendations and thereby adhere to a heart-healthy lifestyle. Mobile phone apps have been emphasized as potential tools to promote preventive activities after attendance in a CR program. Before commencing a trial to assess the potential effect of using an app to promote long-term adherence to preventive activities after attendance in CR, a study to assess if it is feasible to use an app is warranted.

**Objective:**

The goal of the research is to assess if it is feasible to use a mobile phone app for promoting and monitoring patients’ adherence to a heart-healthy lifestyle after CR.

**Methods:**

The study included an experimental, pre-post single-arm trial lasting for 12 weeks. All patients received access to an app aimed to guide individuals to change or maintain a heart-healthy lifestyle. During the study period, patients received weekly, individualized monitoring through the app, based on their own goals. Feasibility outcomes assessed were recruitment rate, adherence to the app, resource requirements, and efficacy regarding capability to detect a change in quality of life, health status, and perceived goal achievement as well as evaluating ceiling and floor effect in these outcomes. Criteria for success were preset to be able to evaluate whether the app was feasible to use in a potential future RCT.

**Results:**

In total, 71% (17/24) of the patients who completed CR were eligible for a potential RCT as well as for this study. All 14 patients included in the study used the app to promote preventive activities throughout the study. Satisfaction with the technology was high, and the patients found the technology-based follow-up intervention both useful and motivational. Ceiling effect was present in more than 20% of the patients in several domains of the questionnaires evaluating quality of life (36-Item Short Form Health Survey and COOP/WONCA functional health assessments) and health status (EQ-5D). Overall self-rated health status (EuroQol Visual Analog Scale) and perceived goal achievement were found to be outcomes able to detect a change.

**Conclusions:**

Individual follow-up through an app after attendance in CR is feasible. All patients used the app for preventive activities and found the app both useful and motivating. Several points of guidance from the patients in the study have been adopted and incorporated into the final design of the RCT now in the field.

## Introduction

Heart disease is the leading cause of death and disability worldwide [1]. Participation in cardiac rehabilitation (CR) is the recommended first step for secondary prevention and is associated with improved prognosis [2,3]. Exercise is the cornerstone in CR, but current guidelines recommend programs that include dietary counseling, optimizing of medical treatment, education, psychological support, and support for smoking cessation [2]. However, it has been shown to be a major challenge for many patients to follow recommendations and thereby adhere to a heart-healthy lifestyle [2,4]. A heart-healthy lifestyle includes regular physical activity, heart-healthy diet, and cessation of tobacco consumption [2]. Only 15% to 50% of individuals attending CR still exercise 6 months after participation, and even less after 12 months [5,6]. Approximately 50% of patients who are smokers prior to a coronary event still smoke 6 months after the cardiac event, and less than 50% of obese patients follow dietary recommendations [7].

Common barriers to adherence to health recommendations after a CR program are lack of social support, patient health beliefs (eg, cause of disease, controllability of a condition), past medical history, and anxiety and depression [8]. To increase adherence, there is a need for long-term individualized follow-up that takes patients’ barriers into account [2,8]. The best way to promote adherence and monitor preventive activities is not known and represents an important knowledge gap in CR [2]. What is known is that the follow-up should use a patient-centered approach that focuses on the patients’ priorities and goals and incorporates lifestyle changes within the context of the patients’ life [2].

Digital health interventions may act as follow-up tools and deliver necessary support for patients either in CR or after attendance in CR [9-11]. Mobile health, or mHealth, defined as the use of mobile computing and communication technologies for health services and information [12], includes many of today’s digital health interventions. Mobile phone apps are considered a particularly promising mHealth tool for secondary prevention for heart patients due to their ability to monitor patients’ health from anywhere at any time [13,14]. As the population becomes more and more technology savvy, apps may appeal to more people. Apps offer advantages to health care providers through access to deliver direct support, interact with patients, and monitor engagement and progress [15]. As such, apps are potential tools for long-term follow-up of patients after attendance in a CR program [9,16]. However, there is limited research on the effect of using an app to promote and monitor adherence to heart-healthy lifestyle after CR. A recent systematic review [17] on the effectiveness of interventions with apps to promote lifestyle changes in patients with noncommunicable diseases found only one study conducted in heart patients [18]. The main outcome was drug adherence, which was significantly better in the intervention group compared with the control group [18].

Randomized clinical trials (RCTs) are needed to assess potential effects of an app that enables individualized monitoring of heart patients after attendance in CR with regard to exercise capacity and other cardiovascular risk factors. Before commencing such a trial, it is necessary to evaluate if it is feasible to use an app for this purpose. The main aim of this study was to assess if a mobile phone app was feasible to use for promoting and monitoring patient adherence to a heart-healthy lifestyle after CR. The following research questions were addressed: To what extent are patients willing to take part in such a study? Will the patients use the app as intended? What resources are needed to deliver follow-up messages and interact with patients? Are the outcomes (questionnaires and self-perceived goal achievements) able to detect a change? The results from this study will guide the design and software in a subsequent RCT.

## Methods

### Study Design

This study was an experimental, pre-post single-arm trial. The evaluation lasted for 12 weeks.

### Setting

The study took place in the eastern part of Norway during spring and early summer 2017. Patients were recruited from two rehabilitation centers. One rehabilitation center offered a 12-week CR program and the other offered 1-week and 4-week programs. Approximately one-third of the participants were recruited from each of the three CR programs for this feasibility trial, the same proportions planned for the upcoming RCT.

### Participants

Eligible patients were women and men over the age of 40 years who completed CR in one of the three programs during a period of two weeks. They had to own and use an Android or iOS mobile phone and be able to read and understand Norwegian or English. Exclusion criteria were restrictions regarding exercise intensity for any reason due to the primary end point in the planned RCT, which is intended to be maximal oxygen consumption (VO_2peak_). Descriptive data collected at baseline included sex, age, diagnosis, treatment, history of smoking, educational level, exercise habits last year, and VO_2peak_.

### Using the App

**Figure 1:**
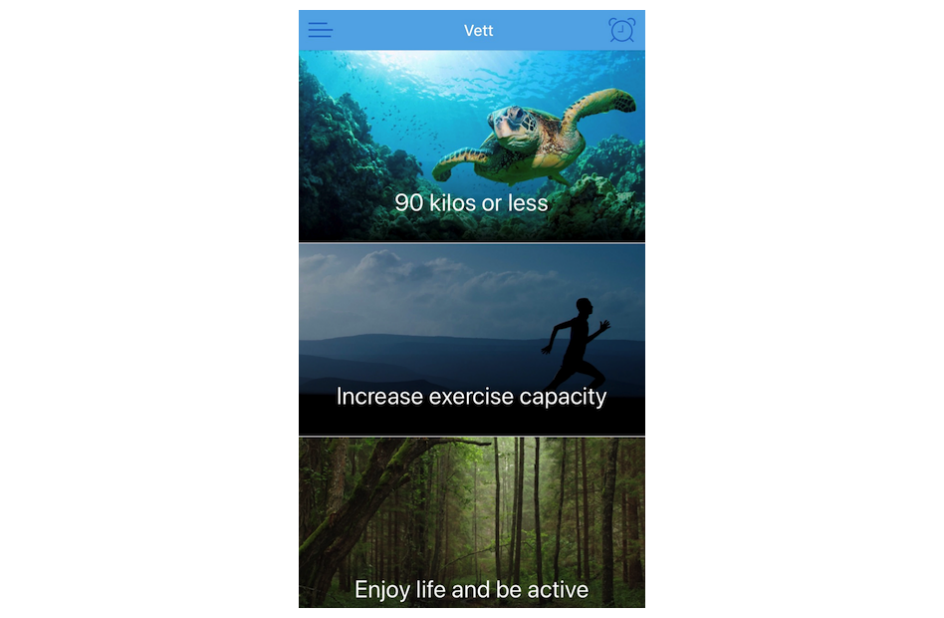
User interface of the app showing individual goals.

**Figure 2:**
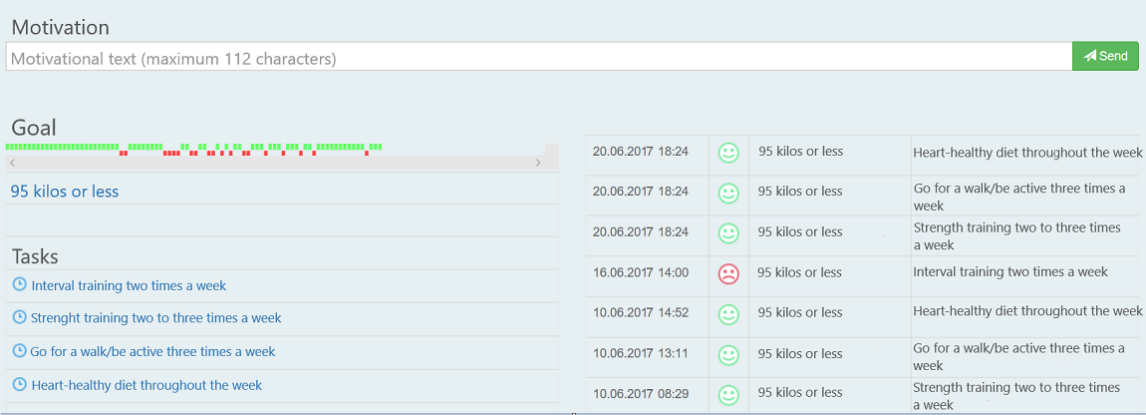
Administrator interface of the app showing one goal with related tasks.

Patients received the app after attendance in CR. The app was developed to guide and help individuals change behavior and maintain habits. The follow-up was based on the transtheoretical model of behavior change [19]. According to this model, health behavior change involves progress through six stages of change: precontemplation, contemplation, preparation, action, maintenance, and termination [19]. In this connection, motivational interviews are used to help people access motivation to change a particular behavior through collaboration, evocation, autonomy, and exploration [20]. The patients are supposed to set goals that are small, important to them, specific, and realistic to achieve [21]. The app used in this study permits the user to create and set such goals (Figure 1) with tasks and accompanying reminders. A supervisor has access to an administrator interface (Figure 2) and can monitor the goals and tasks of each patient. In addition, the patient can write reflections in the app that the supervisor can read in the administrator interface. The app itself provides reminders and evaluations of tasks and weekly goal achievement that automatically pop-up. In these evaluations, the patients must reply with a red or green face depending on whether they have completed the planned tasks or not and rate the weekly goal achievement on a scale from 0 to 100.

At baseline, a supervisor guided the patients in setting individual goals by using elements from motivational interviewing. The supervisor in the study was a physiotherapist specializing in cardiovascular and pulmonary physiotherapy with five years of experience in CR. Each patient was encouraged to set a minimum of two goals with related tasks to be able to reach each goal. The patient decided when and how often reminders of the tasks should appear on their mobile phone. During the follow-up period, the patients received short, tailored, individualized motivational feedback directly through the app 1 to 3 times a week and comprehensive individual feedback through email once a week. Patients could submit questions to the supervisor at any time, receiving an answer within 2 working days. If the question was medically related (eg, changing medication or chest pain), patients were advised to contact their general practitioner. Patients were followed for 12 weeks by the same supervisor who included the patients at baseline.

### Outcome and Measures

#### Recruitment Rate

The proportion of patients willing and able to take part in the study after finishing CR was established. During a 2-week period, all patients at two different centers were invited to participate. Information about restrictions on exercising (exclusion criteria) was collected from health providers at the centers.

#### Adherence to the App

Use of the app was registered in terms of actual use and if patients answered tasks within a week, all based on data shown in the administrator interface (Figure 2). Patient satisfaction with the technology was assessed with the System Usability Scale (SUS), a paper questionnaire completed at the end of the study. The SUS is a technology independent, 10-item questionnaire with a score between 0 and 100 where 0 represents low usability and 100 represents high usability [22]. Patient experiences with the app and follow-up were evaluated through a questionnaire designed for this study consisting of 20 questions; 13 questions with answers on a Likert scale (0 to 100), 5 multiple-choice questions, and 2 open-ended questions (Multimedia Appendix 1). The Likert scale questions allowed patients to evaluate the app with regard to usefulness and motivational effect. The multiple-choice questions provided information about patient satisfaction with follow-up time and frequency of individual feedback. The open-ended questions gave the patients an opportunity to give additional guidance for the upcoming RCT. Any problems with the technology were continuously observed through the administrator interface. Additionally, the supervisor used the app throughout the study to enable early discovery and mitigation of technical issues.

#### Resource Requirements

Throughout the study period, the supervisor logged all time spent monitoring patients.

#### Change and Ceiling and Floor Effect in Outcomes

These outcomes were determined by evaluating whether changes in quality of life, health state, and perceived goal achievement over the 12-week period could be observed and whether these outcomes disclosed ceiling or floor effects. Quality of life was assessed with two questionnaires: the 36-Item Short Form Health Survey (SF-36) and the Dartmouth COOP/World Organization of Family Doctors functional health assessment (COOP/WONCA) [23,24]. The SF-36 consists of 36 questions across eight domains [23]. Item scores were transformed to 0 to 100 point scales (0=worst, 100=best) using the SF-36 syntax [23]. COOP/WONCA consists of six questions across six domains with a score of 1 in each domain representing the best possible score while a score of 5 is the worst possible score [24]. Health status was assessed with EQ-5D [25]. The EQ-5D consists of five questions with five answer options to each question, where a score of 1 is the best possible score and 5 is the worst possible score [25]. In addition, the EQ-5D consists of an overall health question (EQ-VAS) where the patient answers on a Likert scale (0 to 100, where 0 represents the worst possible health and 100 is the best possible health) [25]. All questionnaires were answered by patients on paper at baseline and after 12 weeks. Floor and ceiling effects were considered present in the scales if more than 20% of respondents achieved the lowest or highest possible score, respectively [26]. Therefore, if more than 20% of patients reached floor or ceiling effect, extra emphasis was placed on the evaluation of whether the questionnaire was suitable for the upcoming RCT. Perceived goal achievement was evaluated through the database platform. Every week patients got an automated question in the app—“How close do you think you are to reaching this goal?”—where they would answer on a Likert scale (0 to 100, where 0 represents far away from reaching the goal and 100 that the goal has been reached) for each goal.

### Criteria for Success

In order to determine whether follow-up of patients after CR through an app was feasible in an RCT, we chose the following criteria for success:

- At least 80% of the patients used the app during the study period
- Patients answered at least 50% of the tasks within a week
- Mean SUS score ≥65

### Statistical Analysis and Ethical Consent

Based on Treweeks’ [27] recommendations for pilot and feasibility trials, we needed 10-15 patients to be able to have confidence in the conclusions drawn from the data. Data were analyzed using SPSS Statistics for Windows version 24.0 (IBM Corp). Descriptive statistics are reported for each case and in means and standard deviations for the whole group. Differences in outcome variables (baseline to 12-week) were analyzed using nonparametric tests due to the small number of patients. Significance was set to *P*<.05. In case of missing data, we used the last observation carried forward method.

The Regional Committee for Medical Research Ethics, Region Eastern Norway, reviewed the study and found that approval was not required. All participants provided written informed consent.

## Results

### Recruitment Rate

**Figure 3:**
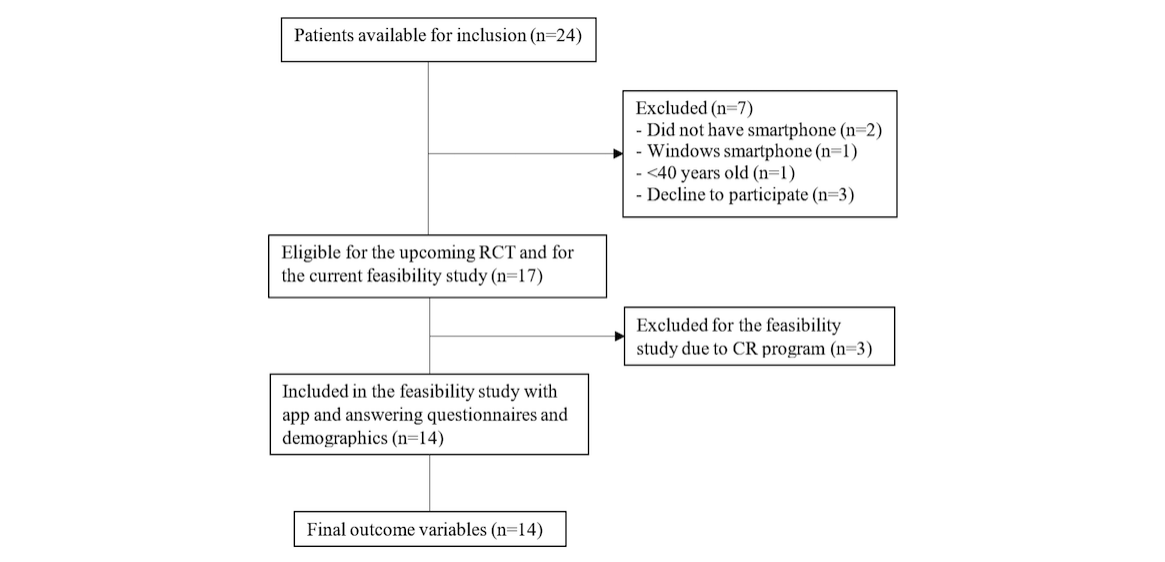
Flow diagram.

In total, 24 patients were available for inclusion in the study (Figure 3), and 17 (71%) were eligible for the potential RCT. Ultimately, 14 patients were enrolled in this study.

Half (50%) of the patients were iOS users, and half were Android users. Approximately one-third had attended each of the three CR programs—12 weeks, 4 weeks, and 1 week. Table 1 provides the baseline characteristics: 71% (10/14) were men, mean age for all participants was 60.1 (SD 8.5) years, and mean VO_2peak_ was 27.6 (SD 6.2) mL/kg/min.

**Table 1:** Baseline characteristics.

Sex	Age	Diagnosis	Treatment	Smoker	Education^a^	Weekly exercise last year^b^	Exercise capacity (VO_2peak_^c^, mL/kg/min)
F^d^	42	ACS^e^	PCI^f^	Earlier	0	3	37.4
M^g^	66	ACS	PCI	Never	5	0	35.6
M	64	ACS	PCI	Earlier	1	1.5	29.4
M	55	CAD^h^	PCI	Earlier	4	0.5	28.1
M	45	ACS	PCI	Never	2	1.5	36.5
F	68	ACS with cardiac arrest	ICD^i^ and medication	Earlier	0	0	16.2
F	62	CAD	PCI	Earlier	5	1	26.6
F	66	Spasm angina	Conservatively	Earlier	0	0	27.6
M	66	CAD	CABG^j^	Earlier	0	0	19.7
M	68	CAD	PCI	Never	7	7	25.6
M	52	CAD	PCI	Never	4	2	28.3
M	66	CAD	Conservatively	Current	3	0	19.7
M	62	CAD and AS^k^	CABG and AVR^l^	Earlier	3	0	28.2
M	60	Atrial flutter	Pacemaker	Never	1	4	27.5

^a^Years of education after high school.

^b^Number of exercise sessions per week lasting at least 30 minutes where participants became sweaty and breathless.

^c^VO_2peak_: maximal oxygen consumption.

^d^F: female.

^e^ACS: acute coronary syndrome.

^f^PCI: percutaneous coronary graft.

^g^M: male.

^h^CAD: coronary artery disease.

^i^ICD: implantable cardioverter defibrillator.

^j^CABG: coronary artery bypass graft.

^k^AS: aortic stenosis.

^l^AVR: aortic valve replacement.

### Adherence to the App

All patients used the app regularly throughout the 12 weeks. Additionally, all patients answered all tasks within a week. Table 2 provides the app use for all patients. All patients had goals or tasks related to exercise or fitness training, and 9 of 14 had goals or tasks related to maintaining or improving their dietary habits. The mean numbers of individual goals, tasks, and weekly reminders were 1.9 (SD 0.5), 3.5 (SD 1.1), and 10.3 (SD 4.5), respectively. The mean SUS score for all patients was 84.8 (SD 12.8). iOS users scored higher in SUS than Android users (90.4 [SD 7.7] vs 79.3 [SD 15.0]) (Mann-Whitney test, *P*=.12), but the difference was not significant. Patients scored 96.8 (SD 7.2) on the questions on usefulness of the app and 91.3 (SD 14.2) on questions regarding their own motivation. Most of the patients (9/14, 64%) reported it to be useful to use the app for 6 to 12 months after attendance in CR. All patients felt it was very important that they were closely monitored by the supervisor during the first months. After that, monitoring could be less frequent.

**Table 2:** App use for all patients.

Sex	Age	Mobile phone model	Number and type of goal	Number of tasks	Number of weekly reminders	SUS^a^ score
F^b^	42	iPhone 5S	Fitness training; Healthy nutrition; Strength training	4	11	90
M^c^	66	iPhone 6S	Fitness training; Physical activity	2	4	80
M	64	Samsung Galaxy S5	Fitness training; Healthy nutrition	3	9	77.5
M	55	Sony Xperia	Fitness training; Healthy nutrition	4	12	62.5
M	45	iPhone 6S	Weight loss; Healthy nutrition	5	17	92.5
F	68	Huawei	Fitness training; Relaxation	4	18	67.5
F	62	iPhone 6S	Fitness training; Mindfulness	5	17	95
F	66	iPhone SE	Fitness training; Overcome anxiety	4	5	95
M	66	Samsung Galaxy S4	Weight loss	4	10	90
M	68	iPhone 6S	Weight loss	2	8	80
M	52	Samsung Galaxy S5	Fitness training; Weight loss	4	7	65
M	66	HTC Sense 6	Smoking cessation; Fitness training	2	8	92.5
M	62	Samsung Galaxy A5	Fitness training; Weight loss	3	8	100
M	60	iPhone 7S	Activity and exercise; Healthy nutrition	4	11	100

^a^SUS: System Usability Scale.

^b^F: female.

^c^M: male.

Only minor problems with the technology appeared during the study. Patients could not report that they had completed tasks for a 9-hour period, and for 4 weeks, patients could not save the score on the weekly perceived goal achievement question that appeared in the app.

### Resource Requirements

The supervisor spent approximately one hour to include each patient to the study. During this hour, the supervisor obtained written consent; collected sociodemographic data; created a user for the app in the administrator interface; helped the patient download the app and set realistic, specific, important, and individual goals and tasks; and trained the patient to use the app. Thereafter, time spent monitoring patients was, on average, 6 minutes per patient per week for the 12 weeks. In addition, on average 7 minutes each week was spent answering patient emails and 9 minutes was spent talking to the service provider about bug fixes and update needs of the app.

### Change and Ceiling and Floor Effect in Outcomes

The domain physical fitness in COOP/WONCA improved from 2.2 (SD 1.0) to 1.9 (SD 0.9), *P*=.046. There were no statistically significant changes in any of the other domains. The domain pain and discomfort in EQ-5D improved significantly, from 2.1 (SD 1.1) to 1.8 (SD 1.1) (Wilcoxon signed-rank test, *P*=.046). There were no statistically significant changes in any of the other domains. On the SF-36, no statistically significant changes were found in any of the domains.

Mean scores with standard deviations for both baseline and 12 weeks with *P* values of the changes are presented in Table 3 in addition to minimum and maximum observed scores and percent of ceiling and floor effects for each questionnaire at baseline, with associated domains.

**Table 3:** Quality of life and health status at baseline and 12 weeks, with *P* values of changes, minimum and maximum scores, and percentages of n reaching floor or ceiling effect at baseline.

Outcome and measure		Baseline mean (SD)	12 weeks mean (SD)	*P* value	Observed (baseline)			
					Min	Max	Floor	Ceiling
**COOP/WONCA^a^ (score 1-5)**								
	Physical fitness	2.2 (1.1)	1.9 (0.9)	.046	1	4	0	36
	Feelings	2.0 (1.0)	1.8 (1.0)	.08	1	4	0	36
	Daily activities	1.7 (0.8)	1.8 (1.0)	>.99	1	4	0	43
	Social activities	1.8 (1.0)	1.6 (0.6)	.32	1	4	0	50
	Change in health	2.4 (1.1)	2.5 (0.9)	.48	1	5	7	21
	Overall health	2.4 (0.9)	2.1 (0.9)	.10	1	4	0	7
**SF-36^b^ (score 1-100)**								
	Vitality	54 (21)	56 (18)	.69	20	85	0	0
	Physical functioning	87 (14)	89 (12)	.64	55	100	0	21
	Bodily pain	67 (25)	67 (29)	.80	22	100	0	14
	General health perception	67 (19)	67 (18)	.61	25	100	0	7
	Physical role functioning	54 (43)	54 (45)	.73	0	100	29	36
	Emotional role functioning	83 (36)	91 (51)	.74	0	100	14	79
	Social role functioning	84 (20)	88 (15)	.30	37.5	100	0	50
	Mental health	76 (17)	74 (14)	.91	48	100	0	7
**EQ-5D^c^ (score 1-5)**								
	Mobility	1.4 (0.9)	1.1 (0.5)	.29	1	3	0	79
	Self-care	1.0 (0)	1.0 (0)	>.99	1	1	0	100
	Usual activities	1.4 (0.6)	1.1 (0.3)	.06	1	2	0	64
	Pain or discomfort	2.1 (1.1)	1.8 (1.1)	.046	1	4	0	36
	Anxiety or depression	1.6 (0.9)	1.6 (0.8)	.79	1	4	0	57
	EQ-VAS^c^	68.9 (11.6)	72 (13.6)	.35	40	93	0	0

^a^COOP/WONCA: Dartmouth COOP/World Organization of Family Doctors functional health assessment chart.

^b^SF-36: 36-Item Short Form Health Survey.

^c^EQ-VAS: EQ-5D Visual Analog Scale.

**Figure 4:**
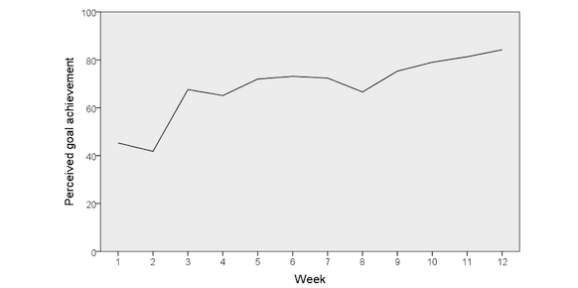
Mean score of perceived goal achievement, week by week.

Mean scores of perceived goal achievement, week by week, are presented in Figure 4. There was a statistically significant improvement in goal achievement from baseline to week 12 with a mean change of 41.2 (SD 39.0) (*P*=.002). None reached ceiling or floor effect. Distribution of scores in COOP/WONCA and EQ-5D at baseline and after 12 weeks are presented in Multimedia Appendix 2 and 3, respectively.

## Discussion

### Principal Findings

To our knowledge, this is the first study to evaluate the feasibility of using an app as a tool to promote and monitor adherence to a heart-healthy lifestyle after attendance in CR with predefined criteria for success. Results demonstrated a high recruitment rate and high adherence to use of the app. In total, 71% of available patients were eligible and wanted to participate in the study, and all patients used the app during the entire intervention period and answered all tasks. The supervisor spent in average of 6 minutes each week to give individualized feedback to each patient. Quality of life, health state, and self-perceived goal achievement improved; however, we observed a ceiling effect in questionnaires measuring quality of life and health status. The strengths of feasibility studies are to report the possible pitfalls of a large RCT, weigh strengths against weaknesses of the intervention, investigate the feasibility of patient recruitment and outcome measures, and come up with solutions on how to conduct the RCT [28]. A strength of our study was its clear eligibility criteria and rigorous protocol, which ensured that the sample included the targeted patient population and accurate data collection at predefined study time points.

Our findings are in line with results from studies evaluating mHealth interventions for chronic disease management. In a systematic review [10], 62 of 107 included studies evaluated usability, feasibility, and acceptability of mHealth interventions. The most used mHealth intervention was text messaging. The number of studies including mobile phone apps is not specified, but it is stated that 25 used specialized software or a mobile phone app. Generally, the review concluded that the usability, feasibility, and acceptability of mHealth tools were high in connection with chronic disease management. Both patients and providers appreciated the mHealth tools [10]. Specific results from studies with mobile phone apps are not presented. In addition, none of the 25 studies that used specialized software or a mobile phone app were conducted in heart patients [10]. A review by our group determined that mobile phone apps seem to be most common in studies conducted in patients with diabetes mellitus [17].

According to the technology acceptance model, usefulness and ease of use are key factors that influence whether people accept or reject technology and thereby influence adherence to a technology-based intervention [29]. In this study, the overall satisfaction with technology measured with the SUS was 84.8. This score is considered as very high with a high degree of usability [30]. There was a difference in SUS score between Android and iOS users in favor of the iOS users. This is not surprising as the iOS operating system has a unified user interface for all mobile phones, whereas the Android operating system comprises several user interfaces due to the wide range of mobile phone producers using the platform. Because of the difference in SUS score between Android and iOS users, it is necessary to make the Android version of the app more stable for the upcoming RCT.

Patients reported clear and realistic goals for a heart-healthy lifestyle that they were able to evaluate weekly in the app. It was surprisingly easy to guide the patients in setting goals. This can be explained because goal-setting is often used as an approach in CR programs [31]. Both CR centers from where the patients were recruited emphasize goal-setting in their CR program. In addition, the supervisor’s background and experience in CR may have contributed to the effective goal-setting. Continuation of focus on their own goals for a heart-healthy lifestyle in the follow-up after attendance in CR may have contributed to patients’ perceived usefulness of the intervention and perceiving it would be useful to be followed up for a longer period of time. Most patients reported that it would be beneficial to use the app for a year. Whether the patients actually preserved or improved their exercise capacity or nutritional-related goals is still uncertain, but the results from this feasibility study support moving on to the RCT.

### From Single-Arm Feasibility to Randomized Controlled Trial

Several points of guidance from the patients in the study have been adopted and incorporated into the final design. First, although satisfaction with the technology was high, some potential improvements were discovered. One to three times a week, individualized motivational messages were sent to each patient. These messages appeared on the patients’ mobile phones as a push notification. It turned out that several of the patients were not familiar with push notifications, and therefore these messages were lost without some of the patients having read the content. Based on the feedback, the app has been adjusted, and individualized motivational messages are saved in the app. Each patient can then decide when they want to read and delete them. A technical problem with weekly goal achievement was fixed during the fifth week of the study, and it is now fully functional. In addition, there were some options in the app with regard to when a task should start. In example, patients could create a task with any start time they wanted. This function did not work properly, and patients reported that they didn’t need it. Therefore, the functionality has been deleted in order to keep the app as simple and easy to use as possible.

Quality of life was evaluated with the questionnaires SF-36 and COOP/WONCA. It turned out that more than 20% of the patients achieved ceiling effect on 50% or more of the domains in these questionnaires, which makes it difficult to detect any improvement in these domains. Floor effect was achieved only in the domain physical role functioning on the SF-36. The high number of patients reaching the upper limits may have been a result of the non–disease-specific questionnaires that were used. Additionally, the included patients were relatively young, not in any acute phase of disease or illness, and had just completed an extensive rehabilitation program [32]. To be able to evaluate possible changes in quality of life in the upcoming RCT, we have decided to use the HeartQoL health-related quality of life questionnaire. HeartQoL has been found to be both valid and reliable in patients with the primary diagnoses the CR patients normally have (eg, angina, myocardial infarction and heart failure [33], stable coronary artery disease [34], atrial fibrillation [35] as well as in patients with implantable cardioverter defibrillators [36] and patients following heart valve surgery [37]). On the EQ-5D, ceiling effect was reached for more than 30% of the patients in all domains. Again, this can be explained by the patients’ relatively young age and the inclusion of nonhospitalized patients [32] and is in line with other research on this population [38,39]. Despite this, we have chosen to keep the EQ-5D in the planned RCT due to its ability to conduct health economic statistics and because HeartQoL doesn’t include an overall health status like EQ-VAS.

In line with the general Norwegian population according to Statistics Norway [40], 92% of the patients at CR were owners and users of mobile phones. Although 71% of the patients were eligible for inclusion in the study and therefore for the upcoming RCT, it is not likely that these patients would sustain participation if they perceived that randomization to the control group resulted in an inferior intervention.

Although the results from this feasibility study are promising for the upcoming RCT, we have to be aware that the patients in the study were only followed for three months, and it is reasonable to believe that there will be dropouts when the study runs over a year. This must be taken into account in the calculation of how many participants will be needed to detect an effect in the RCT, and we have added 20% for possible dropouts.

### Conclusions

Based on preset criteria for success, our study shows that an intervention with an app that allows individualized monitoring after attendance in CR is feasible. All patients used the app to get help for preventive activities such as exercise and dietary change. Implementation of mobile phone apps as a tool to promote adherence to preventive activities after CR is a novel approach. Since research in this area is warranted, this paper may serve as a foundation for other upcoming RCTs as well and inform the development of RCT management.

## Appendix

Multimedia Appendix 1 Questionnaire.

Multimedia Appendix 2 Distribution of scores in COOP-WONCA at baseline (pre) and after 12 weeks (post).

Multimedia Appendix 3 Distribution of scores in EQ-5D at baseline (pre) and after 12 weeks (post).

## Abbreviations

COOP/WONCA: functional health assessment charts developed by the Dartmouth COOP Functional Health Assessment Project and promoted by the World Organization of Family Doctors
CR: cardiac rehabilitation
EQ-5D: standardized measure of health status developed by the EuroQol Group in order to provide a simple, generic measure of health for clinical and economic appraisal
RCT: randomized controlled trial
SF-36: 36-Item Short Form Health Survey
SUS: system usability scale
VO2peak: maximal oxygen consumption
