# Supplementary material for: Feasibility of a Mobile Phone App to Promote Adherence to a Heart-Healthy Lifestyle: Single-Arm Study
Source: JMIR Form Res. 2019 Apr 19;3(2):e12679. doi: 10.2196/12679 (PMC6528433; doi:10.2196/12679)
Supplement: Multimedia Appendix 1 [file formative_v3i2e12679_app1.pdf]

Multimedia Appendix 1. Questions related to your experience with setup and use of the smartphone application Vett®

Based on your experience with Vett®, please rate each statement with a number from 0 to 100. You may think of the number as a percentage, where e.g. the number 100 equals a 100% correct statement in your opinion.

	To which degree do you experience that…	Numeric value (0-100)
1	Vett® on my smartphone motivates me	
2	Vett® on smartphone is simple and intuitive to use	
3	The goal that was set was right for you	
4	The tasks that were registered in the app were right for you	
5	The content of the reminders that Vett® gave you were right for you	
6	Reminders of tasks poped up at the preset time	
7	It is simple and intuitive to answer that reminder has been received	
8	It is simple and intuitive to answer that a task is completed	
9	Weekly self-perceived goal achievement is useful (self-evaluation)	
10	Weekly feedback from supervisor is useful	
	Are there any features missing in Vett®?	

Questions related to the use of Vett® over a longer period

11: Vett® is a useful tool for continued follow-up after participating in cardiac rehabilitation

_______________(0-100)

12: The motivational messages I received in the app were motivating ______________ (0-100)

13: The weekly feedback (e-mail) I received was motivating ______________ (0-100)

14: For how long period would you consider follow-up through an app to be useful after participating in cardiac rehabilitation?

3 months ☐ 6 months ☐ 9 months ☐ 12 months ☐

15: How frequent would you suggest to receive feedback (e-mail) from the supervisor, taken into account your answer on question 14?

Weekly ☐ Biweekly ☐ Every third week ☐ Monthly ☐

Do not want feedback ☐

16: By using Vett® over the period of time suggested on question 14, I would find it motivating to receive motivational messages in the app:

Daily ☐ 2-4 times a week ☐ Weekly ☐ Biweekly ☐ Monthly ☐

17: The first three months I would find it useful to get feedback (e-mail) from supervisor:

Weekly ☐ Biweekly ☐ Every third week ☐ Monthly ☐

Do not want feedback ☐

18: The first three months I would find it motivating to receive motivational messages in the app:

Daily ☐ 2-4 times a week ☐ Weekly ☐ Biweekly ☐ Monthly ☐

Do not want motivational messages ☐

19:

Do you have any other feedback or advice to share, to help improve the usage of an app and follow-up after participating in cardiac rehabilitation?
