# Supplementary material for: Feasibility of a Mobile Phone App to Promote Adherence to a Heart-Healthy Lifestyle: Single-Arm Study
Source: JMIR Form Res. 2019 Apr 19;3(2):e12679. doi: 10.2196/12679 (PMC6528433; doi:10.2196/12679)
Supplement: Multimedia Appendix 2 [file formative_v3i2e12679_app2.pdf]

Multimedia Appendix 2. Distribution of scores in COOP-WONCA at baseline (pre) and after 12 weeks (post), numbers presented in percent (%).

	**No impariments**		**Slightly a little**		**Moderate**		**Quite a bit**		**Extremely**	
	Pre	Post	Pre	Post	Pre	Post	Pre	Post	Pre	Post
**Physical fitness**	36	43	14	21	43	36	7			
**Feelings**	36	50	36	29	21	14	7	7		
**Daily activities**	43	50	50	29		14	7	7		
**Social activities**	50	50	29	43	14	7	7			
	**Much better**		**A little better**		**About the same**		**A little worse**		**Much worse**	
**Change in health**	21	14	36	29	36	50		7	7	
	**Very good**		**Good**		**Fair**		**Poor**		**Very poor**	
**Overall health**	7	21	58	51	21	21	14	7		
