# Supplementary material for: Feasibility of a Mobile Phone App to Promote Adherence to a Heart-Healthy Lifestyle: Single-Arm Study
Source: JMIR Form Res. 2019 Apr 19;3(2):e12679. doi: 10.2196/12679 (PMC6528433; doi:10.2196/12679)
Supplement: Multimedia Appendix 3 [file formative_v3i2e12679_app3.pdf]

Multimedia Appendix 3. Distribution of scores in EQ-5D at baseline (pre) and after 12 weeks (post). Numbers presented in domains is percent (%), number presented in EQ-VAS is mean score.

	**No problems**		**Slight problems**		**Moderate problems**		**Severe problems**		**Extremely problems**	
	Pre	Post	Pre	Post	Pre	Post	Pre	Post	Pre	Post
**Mobility**	79	93	7	7	7		7			
**Self-care**	100	100								
**Usual activities**	64	93	29	7	7					
**Pain/discomfort**	36	57	36	14	14	22	14	7		
**Anxiety/depression**	57	50	36	43			7	7		
	Pre	Post								
**EQ-VAS**	69±12	72±14								
